# THE WHO INTEGRATED CARE FOR OLDER PEOPLE (ICOPE) FRAMEWORK AND THE ASSOCIATION WITH FRAILTY IN OLDER ADULTS

**DOI:** 10.1093/geroni/igae098.3906

**Published:** 2024-12-31

**Authors:** Wan Yun Chou, Su-I Hou

**Affiliations:** University of Central Florida, Orlando, Florida, United States; The University of Central Florida, Orlando, Florida, United States

## Abstract

The World Health Organization (WHO) introduced the Integrated Care for Older People (ICOPE) framework, designed to enhance the intrinsic capacity (IC) of older adults, focusing on their physical and mental health. This shift in care approach, from disease-centered to person-centered, reflects the rapid increase in the global aging population. The objective of the current study was to explore how IC relates to frailty in older adults. In a medical center in Taiwan in 2021, this cross-sectional study involved patients aged 65 and older admitted to the geriatric ward. Participants completed a questionnaire that included an IC assessment based on the six domains of ICOPE (cognition, mobility, nutrition, visual, hearing, and depressive symptoms), Fried’s frailty scale, and demographic information. Fried’s frailty scale categorized participants into robust (score=0), prefrail (score=1-2), or frail (score ≥3) groups. Logistic regression analysis was employed to examine the association between individual ICOPE domains and frailty, adjusting for age, gender, and number of comorbidities. Of the 210 participants, 39.0% were categorized as prefrail and 28.6% as frail. Notably, mobility loss and depressive symptoms were strongly linked to prefrail and frail conditions, with significant adjusted odds ratios (aOR) indicating higher risks. Additionally, malnutrition and hearing loss were significantly associated with frailty. These findings underscore the critical need for attention to IC and frailty in older adults and advocate for regular implementation of the WHO ICOPE screening pathway. While the convenience sampling method may limit generalizability, further research is necessary better to understand the utility of ICOPE in frailty identification.

